# Supporting the use of research evidence in decision-making in crisis zones in low- and middle-income countries: a critical interpretive synthesis

**DOI:** 10.1186/s12961-020-0530-2

**Published:** 2020-02-18

**Authors:** Ahmad Firas Khalid, John N. Lavis, Fadi El-Jardali, Meredith Vanstone

**Affiliations:** 1 Health Policy PhD Program, McMaster University, Hamilton, ON Canada; 2 Department of Health Research Methods, Evidence and Impact, McMaster University, Hamilton, ON Canada; 3 Centre for Health Economics and Policy Analysis, McMaster University, Hamilton, ON Canada; 4 McMaster Health Forum, McMaster University, Hamilton, ON Canada; 5 Department of Political Science, McMaster University, Hamilton, ON Canada; 6 Department of Health Management & Policy, American University of Beirut, Beirut, Lebanon; 7 enter for Systematic Review in Health Policy and Systems Research (SPARK), American University of Beirut, Beirut, Lebanon; 8 Knowledge to Policy (K2P) Center, American University of Beirut, Beirut, Lebanon; 9 Department of Family Medicine, McMaster University, Hamilton, ON Canada

**Keywords:** Critical interpretive synthesis, Health systems research, Barriers, Facilitators, Knowledge translation, Evidence, Decision-making, Crisis zones, LMICs

## Abstract

**Background:**

Decision-makers in crisis zones are faced with the challenge of having to make health-related decisions under limited time and resource constraints and in light of the many factors that can influence their decisions, of which research evidence is just one. To address a key gap in the research literature about how best to support the use of research evidence in such situations, we conducted a critical interpretive synthesis approach to develop a conceptual framework that outlines the strategies that leverage the facilitators and address the barriers to evidence use in crisis zones.

**Methods:**

We systematically reviewed both empirical and non-empirical literature and used an interpretive analytic approach to synthesise the results and develop the conceptual framework. We used a ‘compass’ question to create a detailed search strategy and conducted electronic searches in CINAHL, EMBASE, MEDLINE, SSCI and Web of Science. A second reviewer was assigned to a representative sample of articles. We purposively sampled additional papers to fill in conceptual gaps.

**Results:**

We identified 21 eligible papers to be analysed and purposively sampled an additional 6 to fill conceptual gaps. The synthesis resulted in a conceptual framework that focuses on evidence use in crisis zones examined through the lens of four systems – political, health, international humanitarian aid and health research. Within each of the four systems, the framework identifies the most actionable strategies that leverage the facilitators and address the barriers to evidence use.

**Conclusions:**

This study presents a new conceptual framework that outlines strategies that leverage the facilitators and address the barriers to evidence use in crisis zones within different systems. This study expands on the literature pertaining to evidence-informed decision-making.

## Background

The pressure to demonstrate that responses to crises are grounded in research evidence has been growing over recent years [1–3]. While other domains have been able to make progress in this field, the humanitarian aid domain still faces some challenges [1, 4, 5]. Part of the challenge may be a lack of understanding of the benefits of using evidence to inform decision-making. Research evidence can help decision-makers understand a problem, frame options to respond appropriately, and address implementation considerations for interventions in specific contexts. When used appropriately, evidence can help decision-makers build on the success of others and avoid repeating the failures of others by learning from systematic studies of their impacts and experiences. A significant literature exists that examines the use of research evidence in decision-making, some of which pays particular attention to low- and middle-income countries (LMICs), where most crises occur [6–16]. However, there is a need for a theoretically informed framework outlining the strategies that would leverage facilitators and address the barriers to evidence-informed decision-making in crisis zones in LMICs. This study aims to fill this gap by developing a conceptual framework.

Decision-making is complex, both because it is context dependent and because it is often influenced by the need to act quickly in sometimes less than ideal situations with relatively little access to information. Recognising this complexity, evidence-informed decision-making has been described as an approach that aims to ensure that decisions are influenced by the best available research evidence, while acknowledging the other factors that influence it [17]. These other factors include institutional constraints, interests, ideas such as values, and external factors like the election of a new governing party. In spite of these complexities, strengthening the use of research evidence in decision-making holds the promise of achieving better use of limited humanitarian aid resources.

Crises are no longer contained in one geographical location but rather transcend borders and they can affect mass populations and disrupt health systems. There are several defining characteristics of a crisis situation. First, events that led up to a crisis situation are often unexpected. Second, the crisis event creates uncertainty with what the future holds under this new unexpected event. Third, the crisis event is seen as a threat to the important goals of security and sustainability of a normal structure. Recent humanitarian crises – be it the Ebola epidemic or the Syrian refugee crisis – have placed considerable stress on health systems that are not fully equipped to deal with such crises. For all these reasons, it is important that we start to think how we can build effective humanitarian systems that are able to respond to crises. What makes decision-making in crisis situations unique is the high levels of stress, often in intense and sometimes dangerous situations. Research evidence can help decision-makers respond in a timely manner in such situations.

One area to consider when seeking to strengthen the use of research evidence in crisis zones is what strategies can be used to support evidence-informed decision-making. Up until now, the thinking about the strategies has been mostly confined to the research system, with an emphasis on making evidence more available and accessible to decision-makers and less on formalised processes for facilitating its use [5, 18, 19]. When the focus turns to the humanitarian aid system, the emphasis has been more on establishing a receptive climate for evidence [20]. There has been less attention given to systems beyond the research and humanitarian aid systems. Given the very little research into a fulsome array of strategies to support evidence use in crisis zones, both within and beyond the research and humanitarian aid systems, our compass question is – what are the strategies that leverage the facilitators and address the barriers to evidence use in crisis zones in LMICs? The strategies to support evidence use in crisis zones can be employed to integrate the use of evidence more systematically within different systems.

## Methods

### Design

We used a critical interpretive synthesis (CIS) to develop the theoretical framework and answer our compass question – what are the strategies that leverage the facilitators and address the barriers to evidence use in crisis zones in LMICs? CIS, developed by Dixon-Woods et al. [21], uses many conventional systematic review processes but allows for the examination of both quantitative and qualitative empirical and non-empirical literature (e.g. editorials, essays). This approach is particularly appropriate for this study because there is an ill-defined, diverse, yet nascent body of literature on the barriers to and facilitators of strategies to support evidence use in crisis zones in LMICs. Moreover, contrary to conventional systematic reviews, where there is a well formulated research question at the outset, CIS employs a compass question that allows for a more iterative and responsive process of synthesis as different types of literatures open up new themes and relationships among themes [21, 22].

### Literature search

**Fig. 1:**
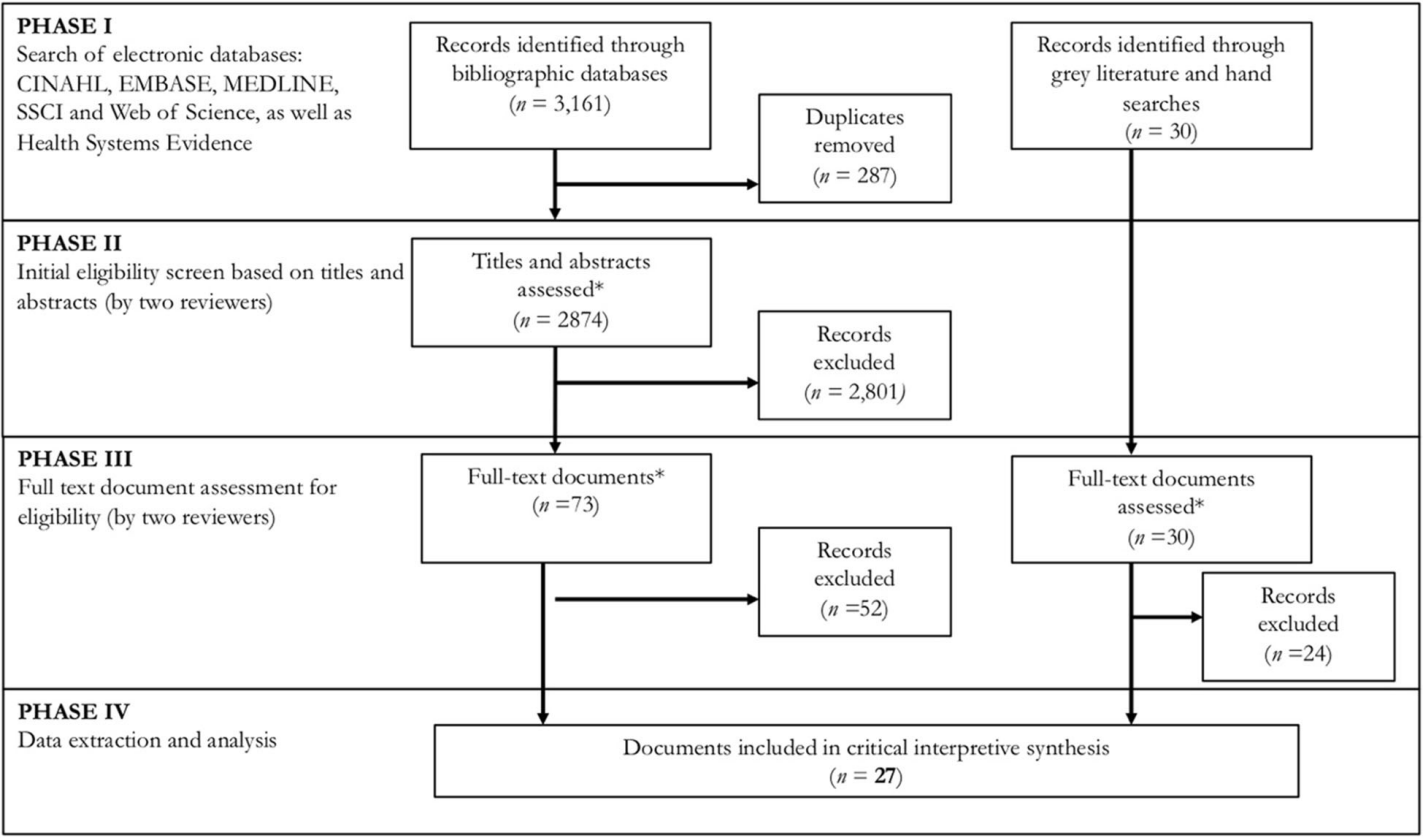
QUORUM flow chart of the inclusion/exclusion process

The literature search was carried out in phases and guided by our compass question and included available research literature that aims, through empirical or non-empirical approaches, to contribute to generalisable knowledge (Fig. 1). Initial search terms were developed in consultation with a librarian (Additional file 1). Several sample search strategies were run and the strategies were adjusted iteratively. Small adjustments were made to the search string for each database to ensure that the formatting is optimal for that database. These database searches were complemented with reviews of the websites of relevant non-governmental organisations (e.g. Médecins Sans Frontières) and international agencies (e.g. WHO), and a hand search of reference lists from relevant articles. The searches were executed from February to April 2017, with additional articles added throughout the analysis phase to fill any conceptual gaps. Duplicate articles resulting from the above parameters were excluded using the EndNote database.

### Article selection

For inclusion, the documents had to provide examples of strategies, facilitators and/or barriers to evidence use in crisis zones in LMICs. For the purpose of article selection, we defined research evidence as the output of research that has been conducted in a systematic way and reported in a transparent manner. Our definition of research evidence includes evidence described in both empirical papers (e.g. observational studies, surveys and case studies) and conceptual papers (e.g. theoretical papers). It also includes both primary studies and secondary research (e.g. systematic reviews and other forms of evidence synthesis). We distinguish such research evidence from other types of information, including data, tacit knowledge or ordinary knowledge [23], and stakeholder opinions.

We excluded the following types of articles: (1) focused on translating clinical research into practice; (2) focused on translating health knowledge to citizens (e.g. patients, members of the public); (3) focused on information systems that deal with raw data and not research evidence; and (4) deemed to be fatally flawed (as determined by an adapted version of the criteria proposed by the National Health Service National Electronic Library for Health for the evaluation of qualitative research, which assess the appropriateness of the aims and objectives and of the research design, etc.).

We assessed the relevance of included studies in the synthesis. For the purposes of this interpretive review, we applied a low threshold of relevance to maximise the inclusion and contribution of a wide variety of papers that address the objectives of this synthesis [24]. We did not perform an appraisal of quality because the core objective is the development of a theoretical framework based on insights and interpretation drawn from relevant sources, rather than those that meet particular quality criteria.

A second reviewer (KM) was assigned to a representative sample of articles to ensure intercoder reliability at two stages of article selection (e.g. titles and abstracts and full-text documents). Given that this is a mixed method synthesis, a Cohen’s Kappa statistic measuring inter-rater agreement was performed with the intent of spurring reflection about the inclusion and exclusion criteria for this study rather than being overly focused on the quantitative estimate [25]. As a result of that reflection, we developed a working dictionary of key terms to be used in the synthesis (e.g. knowledge vs. research evidence). Discrepancies were identified and resolved through discussion.

Similar to a grounded theory approach, additional articles were purposively sampled from the broader literature providing insight into strategies to support evidence use in other settings but that are equally relevant to crisis zones [26]. The additional articles helped with the interpretive process that led to our conceptual framework.

### Data synthesis and analysis

All included papers (*n* = 27) were read in full and any specific information in the results and discussion sections of the included papers that shed light into the topic area were considered as data. The overarching guide used when developing categories for data synthesis was that the category contributed to answering our compass question. Concepts that were repeated in papers that do not provide a new insight into the topic area were excluded as the focus was on uncovering new insights into the strategies to support evidence use, and the facilitators of and barriers to evidence use in crisis zones.

Facilitators and barriers to evidence use were identified if they were referenced in the original text. Strategies were identified for this synthesis in three ways. First, strategies were identified if they were explicitly referenced in the original text. Second, strategies were deduced and extrapolated based on the implications of the identified facilitators and barriers in the literature and the principal investigator’s accumulated understanding of the knowledge translation field. Third, strategies were drawn from the broader literature providing insight into strategies to support evidence use in other settings but that are equally relevant to crisis zones. For example, strategies were drawn from the Lavis et al. [27] framework for assessing country-level strategies to link research to action and the Cochrane Knowledge Translation Strategy framework [27, 28].

An interpretive analytic approach was used to synthesise the results and help develop the conceptual framework. We used a constant comparative method throughout the analysis where emerging data was compared to previously collected data to find similarities and differences [26, 29]. This approach included observations on the concepts used to describe the strategies that leverage the facilitators and address the barriers to evidence use within each system. All data collected were reviewed and detailed notes of the concepts that emerged were included in the analysis.

## Results

### Included articles

All 27 documents selected were published between 2002 and 2017 (Table 1). The region of focus for all documents was LMICs, with a wide range of country of focus (e.g. India, Peru, South Africa). Of the 27 documents, 16 focused solely on natural hazards (e.g. tsunami), 5 on man-made hazards (e.g. armed conflict), and 6 on both. The Cohen’s Kappa was 0.78 for the initial eligibility screen based on titles and abstracts and it was 0.87 for the full-text document assessment, both of which are considered as excellent inter-rater agreement [56]. Five articles were deemed fatally flawed and thereby excluded from our results.

**Table 1:** Characteristics of included studies retrieved in searches and with additional purposive sampling

Year	Country/region of focus	Hazard type	Crisis	Reference
2002	Democratic Republic of Congo	Man-made hazard	Refugee crisis	[30]
2005	Southeast Asia	Natural hazard	Tsunami	[31]
2005	Grenada	Natural hazard	Hurricane	[32]
2008	Southeast Asia & China	Natural hazard	Tsunami, earthquake	[33]
2009	LMICs	Man-made hazard	Armed conflicts	[34]
2010	Southeast Asia	Natural hazard	Tsunami	[35]
2011	LMICs	Man-made and natural hazards	Multiple	[36]
2012	Southeast Asia	Natural hazard	Tsunami	[37]
2012	Peru, Uganda, Nepal	Man-made and natural hazards	Peru: Earthquake, tsunami, Uganda: Armed conflict, Nepal: Floods, refugee crisis, armed insurgency	[38]
2014	Haiti	Natural hazard	Earthquake	[39]
2014	LMICs	Natural hazard	Earthquakes, fires, and floods	[40]
2014	LMICs	Man-made and natural disasters	Multiple	[41]
2014	LMICs	Man-made and natural disasters	Natural disaster, industrial disaster, chemical/biological/radiological/nuclear, conflict, terrorism, civil disturbance, outbreaks, epidemics, pandemics, major transport accidents, generic, multiple, other	[42]
2014	India	Natural hazard	Earthquakes, drought, cyclone, tsunami	[43]
2015	Southeast Asia	Natural hazard	Tsunami	[44]
2015	India	Natural hazard	Flu pandemic	[45]
2015	Southeast Asia	Natural hazard	Tsunami	[46]
2015	Pakistan & Haiti	Natural hazard	Floods, earthquake	[47]
2015	Zimbabwe	Natural hazard	droughts	[48]
2015	LMICs	Man-made hazard	Tsunami, refugee crisis	[49]
2015	South Africa	Natural hazard	Floods, wildfires, droughts, storm waves	[50]
2016	East Africa	Man-made and natural disasters	Conflict, draught, famine, internally displaced person, and refugee crisis	[51]
2016	Nepal	Natural hazard	Lightning strikes, floods, earthquakes and landslides	[52]
2016	South Africa	Natural hazard	Floods, droughts, storm waves and wildfires	[53]
2016	LMICs	Man-made hazard	Fragile and conflict-affected states	[54]
2017	LMICs	Man-made and natural hazards	Armed conflicts and natural disasters	[1]
2017	LMICs	Man-made hazard	Fragile and conflict-affected states	[55]

### Four-part structure of the framework

**Fig. 2:**
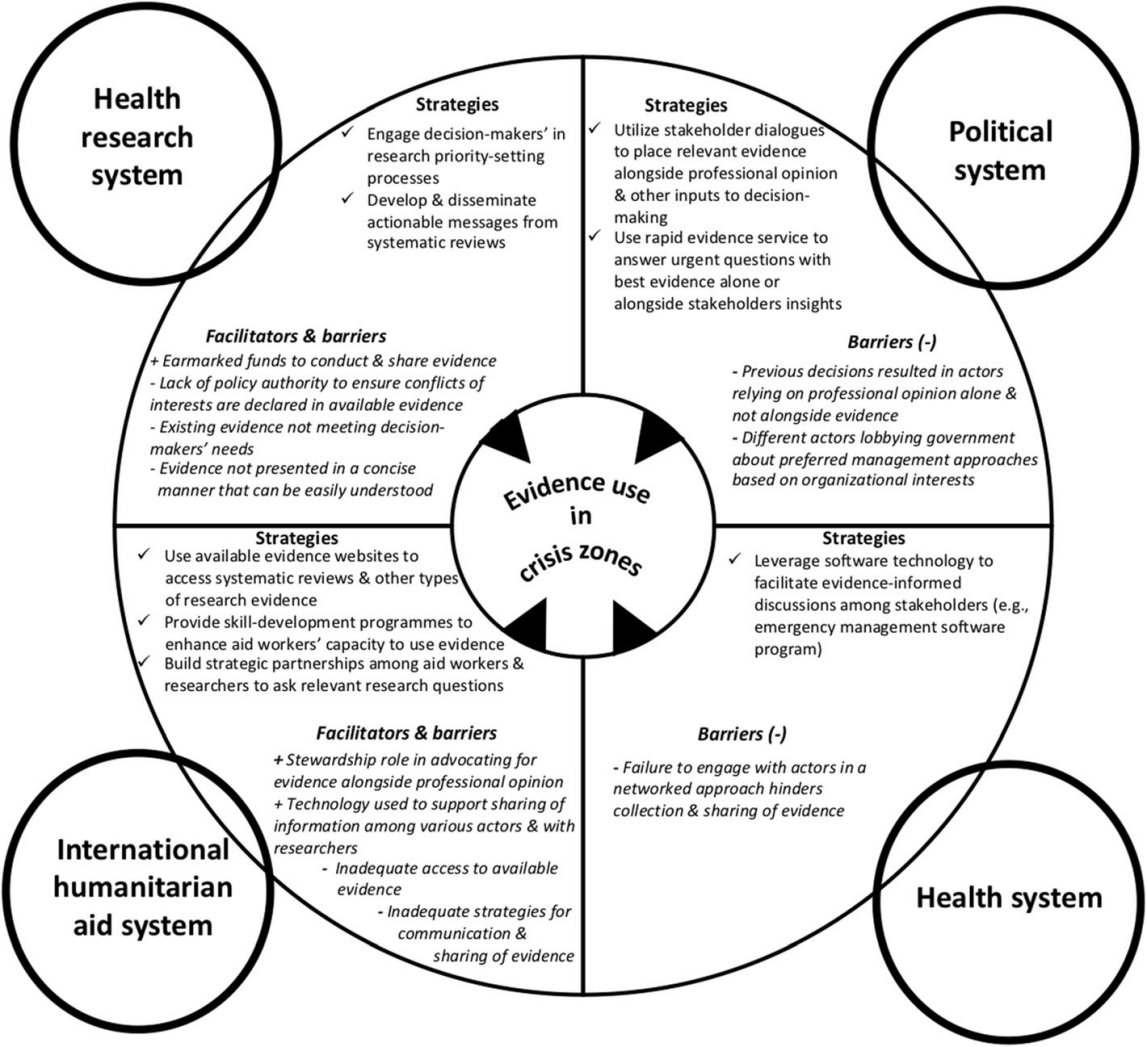
Strategies and the facilitators (+) and barriers (−) to support evidence use in crisis zones

Our analysis of the findings from the literature resulted in a conceptual framework (Fig. 2) that focuses on evidence use in crisis zones examined through the lens of four distinct systems that crisis zones operate within (i.e. political, health, international humanitarian aid and health research). The political system refers to the various actors at the government level tasked with setting laws that pertain to the health, international humanitarian aid and health research system. For the political system, the two main domains consists of institutional constraints and different actors interests influencing evidence use, informed through the 3-I framework – a political science framework with three categories of influences on the policy-making process, namely ideas, interests and institutions [57].

The health system refers to Ministries of Health and health organisations that, when well-functioning, are able to get the right programmes, services and drugs to those who need them. The international humanitarian aid system refers to organisations that are involved in delivery of humanitarian aid services. Some of the principles of the humanitarian aid system that guide interventions in crisis zones include focusing on the most vulnerable population first and operating with impartiality, independence, neutrality, etc. The health research system refers to the people and organisations engaged in the conduct, synthesis and dissemination of research [58]. For the health, international humanitarian aid and health research systems, the facilitators and barriers were analysed according to arrangements that were informed through an established health systems taxonomy that includes governance (i.e. who can make what types of decisions to support evidence use), financial (i.e. understanding how funds can be channelled in ways that support evidence use) and delivery (i.e. infrastructure to support evidence use) [59]. Within each of the four systems, the framework identifies the most actionable strategies that leverage the facilitators and address the barriers to evidence use.

Table 2 outlines, in more detail, the facilitators of and barriers to evidence use in crisis zones in LMICs and the strategies aimed at specific actors within each system to support evidence use. Below, we provide our interpretation about the strategies that leverage the facilitators and address the barriers to support evidence use in decision-making in crisis zones, recognising that many of them are transferable across other applicable systems.

**Table 2:** Strategies and the facilitators (+) and barriers (−) to support evidence use in crisis zones

System & domain		Facilitators (+) of and barriers (−) to research evidence use in crisis zones in LMICs
Political system	Institutional constraints	• Policy legacies: (–) Previous decisions based on experience and opinions because of perceived lack of existing research evidence within the national disaster management system resulted in an interpretive effect among the various actors involved in the delivery of humanitarian aid to rely heavily on professional opinion to inform their decision-making instead of also using existing research evidence to clarify a problem, frame options and address implementation considerations alongside other factors that influence decision-making [30, 41, 43, 51, 52]
	Interests	• Societal interest groups: (–) Different actors lobbying government about preferred disaster management approaches based on organisational interests instead of using existing research evidence to clarify a problem, frame options and address implementation considerations alongside other factors that influence decision-making[51, 52]
		Strategies aimed at policy-makers to support evidence use
		✓ Utilise stakeholder dialogues to place relevant evidence alongside professional opinion and other inputs to decision-making[14] ✓ Use rapid evidence service to answer urgent questions with best available evidence alone or alongside stakeholders’ insights
Health system	Governance arrangements	• Stakeholder involvement and on what terms: (–) Failure to engage with appropriate groups, in a system that has adopted a networked approach to delegating tasks with humanitarian aid delivery, hinders the collection and sharing of evidence [1, 34, 38, 51, 55]
		Strategies aimed at health-system leaders to support evidence use
		✓ Leverage software technology to facilitate evidence-informed discussions among stakeholders (e.g. emergency management software programme) [60, 61]
International humanitarian system	Governance arrangements	• Organisational decisions to support evidence-use: (+) Stewardship role in advocating that existing evidence alongside professional judgement can help inform decision-making about humanitarian responses [1, 30, 39–41, 51, 52, 54, 55]
	Delivery arrangements	• Supports used to assist those receiving evidence: (–) Inadequate access to available evidence (e.g. requires payment, evidence scattered across reports and journals) [31, 34, 35, 39–42, 45, 51, 55] (–) Inadequate strategies used for communication and collaboration among aid workers and researchers to understand and address their knowledge needs [39, 40, 51, 52, 55] (–) Inadequate strategies used to share evidence among multi-institutional humanitarian aid organisations and the network of government level stakeholders [1, 38–41, 47, 50, 51, 53, 55] (+) Technology, such as social networking capabilities (e.g. Twitter, LinkedIn), is used to support the sharing of information among the various actors involved in the delivery of humanitarian aid and with researchers addressing the knowledge needs of aid workers [39, 51]
		Strategies aimed at humanitarian aid decision-makers to support evidence use
		✓ Use available evidence websites to access systematic reviews and other types of research evidence [1, 39, 40, 51, 54, 55] ✓ Provide skill-development programmes to enhance aid workers’ capacity to understand and use research studies [1, 38, 47, 50] ✓ Build strategic partnerships among aid workers and researchers to ask relevant research questions [38, 39, 41, 47, 50]
Health research system	Governance arrangements	• Policy authority: (–) Lack of policy authority to ensure that all personal, organisational and political party-related conflicts of interest are declared in available research evidence by researchers [35, 38, 41]
	Financial arrangements	• Funds to: (+) Conduct research to fill gaps in existing research evidence in a timely manner (e.g. earmarked funds to conduct research in specific crisis zones to address key knowledge gaps) [1, 30, 31, 34, 36, 51, 54, 55] (+) Share research evidence (e.g. earmarked funds for dissemination of research evidence) [41]
	Delivery arrangements	• Enabling use of evidence: (–) Existing evidence not meeting decision-makers’ needs (e.g. lacks implementations considerations for interventions) [1, 33, 35, 38, 40–44, 46–49, 51, 52, 54, 55] (–) Evidence not presented in a concise manner that can be easily understood by non-technical decision-makers [30, 31, 34, 36, 38, 39, 41, 43, 44, 47, 51, 52]
		Strategies aimed at research producers to support evidence use
		✓ Engage decision-makers’ in research priority-setting processes to develop specific research questions related to humanitarian action in crisis zones [33, 34, 38–41, 51, 54, 55, 62] ✓ Develop and disseminate actionable messages for decision-makers, particularly by research organisations that produce syntheses or systematic reviews [63]

### Strategies, facilitators and barriers in each section of the framework

#### Political system

Policy-making about the health, international humanitarian aid and research systems have historically drawn heavily on professional opinion [30, 41, 43, 51, 52]; this reliance on professional opinion is attributed to two main factors. First, decision-makers perceive a lack of existing research evidence to clarify problems, frame options and address implementation considerations. Second, decision-makers need research evidence presented to them alongside other factors that influence their decisions (e.g. stakeholders’ opinions and citizens’ values). Relying solely on professional opinion comes with potential associated errors [64]. For example, cognitive bias is a type of error in thinking that stems from our inability to be entirely objective, resulting in inaccurate judgement. This is not to say that professional opinions should not be highly valued, but rather that it has to be considered alongside the existing research evidence to minimise associated errors.

There are at least two strategies that policy-makers can draw upon to address the barrier of research evidence not being presented alongside other factors that influence decision-making. First, stakeholder dialogues aim to place relevant evidence alongside professional opinion [65]. This strategy is better suited to a protracted crisis as it requires time to prepare an evidence brief to inform the dialogue and adequate resources to support this type of collective problem-solving (e.g. infrastructure needed to convene the dialogue participants). Policy-makers should consider whether they or another group are better positioned to produce the evidence briefs and conduct the policy dialogues. For example, the Knowledge to Policy (K2P) Center in Beirut produced evidence briefs and conducted policy dialogues over a 6-month period to support evidence use in the country’s response to the Syrian refugee crisis [66, 67]. For a fast-evolving crisis, a rapid evidence service can answer an urgent question with the best available evidence alone or alongside insights from key stakeholders (drawn from key-informant interviews) in a short time-frame [68].

#### Health system

The barriers to the use of evidence at the health system level deal mostly with key stakeholders’ involvement with the health services element of humanitarian aid delivery. Stakeholder involvement serves two purposes in supporting evidence use in crisis zones [1, 34, 38, 51, 55]. First, it allows for sharing of evidence among the appropriate groups in a system that has adopted a networked approach to delivering health services as part of humanitarian aid. Second, it strengthens “*local ownership of research*”, which facilitates better uptake of evidence [51]. For example, the Lebanese health system during the Syrian refugee crisis established networks with key stakeholders to collect and share relevant evidence and other types of information to better address the health needs of Syrian refugees [69].

To address challenges with stakeholder involvement and given the dynamic environment of crises, it is imperative for health system leaders to invest in building partnerships with key stakeholders involved in the delivery of the health services element of humanitarian aid to improve evidence sharing and use [50, 51, 53]. One way to build this partnership is by leveraging technology to facilitate evidence-informed discussions among stakeholders. For example, a National Emergency Management Network was created after Hurricane Katrina, which is basically an emergency management software programme that provides a common platform with other participants to share relevant information [60, 61].

#### International humanitarian aid system

Creating new evidence is a costly and time-consuming strategy. A recent estimate found that there are more than 200,000 systematic reviews across all topic areas, although only a small fraction of these reviews are related to humanitarian aid [70]. Undoubtedly, there will always be gaps that need filling in the existing evidence on humanitarian action [33, 52]. However, there is an abundance of existing evidence that is not being used by humanitarian aid workers because of access barriers (e.g. payment required to access evidence, evidence scattered across reports and journals) [31, 34, 35, 39–42, 45, 51, 55].

Evidence websites do exist and can help to address the barriers related to access to systematic reviews. For example, the Evidence Aid website collates systematic reviews specifically aimed at humanitarian action [32]. However, there is a need to increase awareness among humanitarian aid workers on the existence of such sites and their added value in supporting evidence use in decision-making [1, 39, 40, 51, 54, 55]. Humanitarian aid organisations can host training workshops that can be customised to address decision-makers evidence needs in crisis zones. Additionally, decision-makers can enrol in online courses designed to help them find and use research evidence to inform their decision-making (e.g. McMaster Health Forum Finding and Using Research Evidence to Inform Decision-Making in Health Systems and Organizations).

#### Health research system

Supporting the use of healthcare research in decision-making is a complex process that both researchers and decision-makers in crisis zones struggle with [71]. Many authors emphasised that part of the struggle is that existing evidence does not meet decision-makers’ needs (e.g. evidence about interventions does not address implementation considerations) and that the evidence is not presented in a concise manner that can be easily understood by non-technical decision-makers [1, 30, 33–35, 38–44, 46–49, 51, 52, 54, 55, 72, 73].

The research literature on the best strategies to support the use of research evidence in decision-making suggests that interactive engagement between researchers and decision-makers may be most effective [63]. For example, decision-makers can be engaged in research priority-setting processes to develop specific research questions related to humanitarian action in crisis zones [33, 34, 38–41, 51, 54, 55, 62, 74]. Another key strategy is to develop and disseminate actionable messages for decision-makers, particularly by research organisations that produce syntheses or systematic reviews, not single studies. Systematic reviews “*focus on bodies of research knowledge*” that are critical to the development of actionable messages [63]. Knowledge brokers can fill the gap by acting as ‘intermediaries’ between the world of research and decision-making, helping to turn research findings into actionable messages to support their use in crisis zones [38, 53, 55, 75–77].

## Discussion

Our theoretical framework can be thought of as a heuristic that can be used to identify (1) the strategies that can be employed to integrate the use of evidence more systematically into decision-making as well as (2) the facilitators and barriers that influence evidence use in decision-making in crisis zones, both individually and in relation to each other (Fig. 2). The different strategies can be undertaken by different actors within each system – political, health, humanitarian aid and research – that have an influence on the use of evidence in crisis zones. The strategies to support evidence use can occur sequentially or simultaneously within or across the four systems. Our conceptual framework offers a window into the continued progress regarding both the conceptual and practical implementation of strategies to support evidence use in decision-making in crisis zones.

Discussion around the use of evidence in humanitarian action has been ongoing since the 1990s, but much of the discussion has been around filling the knowledge gaps by conducting new research in crisis zones. Our review recognises that there are times when the existing research evidence on crisis zones is lacking (e.g. crisis-specific facilitators of and barriers to the implementation of interventions) and rapid operational research is needed. However, strategies are needed to support the use of the vast pool of high quality and locally applicable research evidence. For example, an organisation has collected such evidence in a freely available online resource (e.g. Evidence Aid).

The focus in the broader literature has been on emphasising the importance of research evidence, even as it acknowledges that research evidence is only one input into the decision-making processes [78–81]. This is especially problematic in the humanitarian aid sector where professional judgement is known to play a key role in informing decisions [1, 70, 82]. Our review recognises that decisions are not determined by evidence alone, but rather alongside professional opinion and other inputs to decision-making. This is why in the political system, we proposed strategies such as stakeholder dialogues that allow the research evidence to put alongside the tacit knowledge and real-world views and experiences of front-line staff [83].

The broader literature contains many strategies to support evidence-informed decision-making in other settings that are equally relevant to crisis zones [20, 28, 65, 76, 83–85]. For example, in healthcare settings, rapid evidence summaries have emerged as a responsive approach involving the presentation of short summary of evidence from systematic reviews, making them more useful and easier to take in by decision-makers [86]. Rapid evidence summaries can also be useful in the humanitarian aid sector, given the need for evidence to be presented in a concise manner that can be easily understood by non-technical decision-makers in a short time-frame [30, 31, 34, 36, 38, 39, 41, 43, 44, 47, 51, 52].

### Strengths and limitations

The strengths of the study included the use of a critical interpretive synthesis methodology that harnessed both a rigorous traditional systematic review methodology with the benefits of an interpretive approach (e.g. evolving compass question, purposive sampling of a diverse literature). Additionally, a second reviewer was involved in the two phases of article selection and in the inclusion phase and a Cohen’s Kappa statistic was completed, with a result that indicated excellent inter-rater agreement and spurred reflection about the appropriate inclusion and exclusion of articles. Finally, the synthesis identified the strategies to support evidence use and the facilitators of and barriers to evidence use, within different systems, that can serve as a point of departure for researchers undertaking empirical work that focuses on one or more specific systems.

Within humanitarian aid research, this study is the first to explicitly focus on the four interconnected systems – political, health, international humanitarian aid and health research. Research to date has tended to take a broader, non-system-specific approach to examining evidence use in crisis zones. This makes it challenging to identify which system the strategies to support evidence use are best handled by and, within a system, which actor is best suited to implement the strategies. The systems level analysis explored in this study contributes to alleviating this challenge by focusing on each system specifically and the actors that can exert influence on supporting evidence use within them.

Despite the merits of our approach, a limitation of the study was that, at times, it was difficult to know from the literature which system the strategies to support evidence use in crisis zones are best handled by and, within a system, whether the strategies are focused on policy-makers, health-system leaders, humanitarian aid decision-makers or research producers. In addition, literature stemming from highly insecure contexts was less available as often researchers have difficulty conducting research in such settings. We addressed these limitations by drawing on existing knowledge translation literature to inform our interpretation of those who would be best positioned to support evidence use, and by suggesting strategies that can be applicable in highly insecure contexts (e.g. rapid evidence service).

In addition, despite our best efforts to examine evidence use in crisis zones, we were unable to make assertions on how context influences the application of strategies to support evidence use in crisis zones in different systems. For example, it is considerably easier to convene a stakeholder dialogue to inform policy options within a relatively stable county (i.e. for Syrian refugees in Lebanon), rather than attempting to convene dialogue in the midst of war zones, outbreaks or natural disasters. However, the findings presented in this study serve as a foundation for research that aims to explore the impact of context on strategic outcomes related to evidence use.

### Implications for policy and practice

The results of our study may enable different actors in crisis zones to reflect on how they can utilise their professional position to support the use of evidence in decision-making, both in the system within their sphere of at least potential control and in the other systems that may be within their sphere of influence. For example, policy-makers in the political system can engage researchers in the health research system to help facilitate a stakeholder dialogue. We recognise that asking these actors to adopt or adapt established strategies and develop new ones that address all the barriers and leverage all of the facilitators is a big challenge to undertake. Our hope is that our framework and strategies serve as the starting point for incremental change to occur over time with the goal of getting closer to addressing the evidence needs of decision-makers in crisis zones.

### Future research

Future studies could apply our theoretical framework in purposively sampled crises, examining specific facilitators of and barriers to research evidence use in decision-making as well as which strategies, if any, are used to leverage the facilitators or address barriers. This would be beneficial in drawing lessons from the framework’s application and in identifying gaps in the framework that need to be addressed. Additionally, future studies could apply the strategies in one or more of the four involved systems to examine whether and how they increase the prospects for evidence use in crisis zones. This could potentially better inform the design of future strategies to support the use of research evidence in such situations and contribute further to our understanding of what types of influence each strategy could be expected to have if successfully implemented in different systems and for different types of crises.

## Conclusions

During a humanitarian response, decision-makers tend to rely on their professional judgement to make decisions as their main goal is the provision of support to people affected by the crisis in often unpredictable situations. Part of the challenge in getting decision-makers to account for research evidence alongside their professional judgement is their uncertainty of whether the existing research evidence can be applied to their unique setting. What is currently missing from the theory is specific strategies to support evidence use in crisis zones that leverage the facilitators and address the barriers to evidence use within different systems (e.g. political, health, etc.). This study offers a new conceptual framework that addresses this gap by identifying and helping to explain the strategies that can be employed to integrate the use of evidence more systematically in crisis zones.

## Supplementary information

**Additional file 1: Table S1.** Initial database search strategy.

## Data Availability

Not applicable.

## Abbreviations

CIS: Critical interpretive synthesis
LMICs: Low- and middle-income countries

## References

[1] Blanchet Karl, Ramesh Anita, Frison Severine, Warren Emily, Hossain Mazeda, Smith James, Knight Abigail, Post Nathan, Lewis Christopher, Woodward Aniek, Dahab Maysoon, Ruby Alexander, Sistenich Vera, Pantuliano Sara, Roberts Bayard (2017). Evidence on public health interventions in humanitarian crises. The Lancet.

[2] Dijkzeul D, Hilhorst D, Walker P (2013). Introduction: evidence-based action in humanitarian crises. Disasters.

[3] Ager A, Burnham G, Checchi F, Gayer M, Grais RF, Henkens M (2014). Strengthening the evidence base for health programming in humanitarian crises. Science.

[4] Banatvala N. (2000). Conflict and health: Public health and humanitarian interventions: developing the evidence base. BMJ.

[5] Turner T, Green S, Harris C (2011). Supporting evidence-based health care in crises: what information do humanitarian organizations need?. Disaster Med Public Health Prep.

[6] Innvær S, Vist G, Trommald M, Oxman A (2002). Health policy-makers' perceptions of their use of evidence: a systematic review. J Health Serv Res Policy.

[7] Harries U, Elliott H, Higgins A (1999). Evidence-based policy-making in the NHS: exploring the interface between research and the commissioning process. J Public Health.

[8] Orton L, Lloyd-Williams F, Taylor-Robinson D, O'Flaherty M, Capewell S (2011). The use of research evidence in public health decision making processes: systematic review. PLoS One.

[9] Lavis JN, Ross SE, Hurley JE (2002). Examining the role of health services research in public policymaking. Milbank Q.

[10] Jewell CJ, Bero LA (2008). “Developing good taste in evidence”: facilitators of and hindrances to evidence-informed health policymaking in state government. Milbank Q.

[11] Sauerborn R, Nitayarumphong S, Gerhardus A (1999). Strategies to enhance the use of health systems research for health sector reform. Tropical Med Int Health.

[12] Cheung A, Lavis JN, Hamandi A, El-Jardali F, Sachs J, Sewankambo N (2011). Climate for evidence-informed health systems: a print media analysis in 44 low-and middle-income countries that host knowledge-translation platforms. Health Res Policy Syst.

[13] Hyder AA, Corluka A, Winch PJ, El-Shinnawy A, Ghassany H, Malekafzali H (2010). National policy-makers speak out: are researchers giving them what they need?. Health Policy Plan.

[14] Pappaioanou M, Malison M, Wilkins K, Otto B, Goodman RA, Churchill RE (2003). Strengthening capacity in developing countries for evidence-based public health:: the data for decision-making project. Soc Sci Med.

[15] Varkevisser CM, Mwaluko GM, Le Grand A (2001). Research in action: the training approach of the Joint Health Systems Research Project for the Southern African Region. Health Policy Plan.

[16] Shroff Z, Aulakh B, Gilson L, Agyepong IA, El-Jardali F, Ghaffar A (2015). Incorporating research evidence into decision-making processes: researcher and decision-maker perceptions from five low-and middle-income countries. Health Res Policy Syst.

[17] Oxman AD, Lavis JN, Fretheim A, Lewin S (2009). SUPPORT Tools for evidence-informed health Policymaking (STP) 17: Dealing with insufficient research evidence. Health Res Policy Syst.

[18] Kayabu B, Clarke M. The use of systematic reviews and other research evidence in disasters and related areas: preliminary report of a needs assessment survey. PLoS Curr. 2013;5. 10.1371/currents.dis.ed42382881b3bf79478ad503be4693ea 10.1371/currents.dis.ed42382881b3bf79478ad503be4693ea PMC3556506 23378935

[19] Pringle JD, Cole DC (2009). Health research in complex emergencies: A humanitarian imperative. J Acad Ethics.

[20] Ellen ME, Léon G, Bouchard G, Lavis JN, Ouimet M, Grimshaw JM (2013). What supports do health system organizations have in place to facilitate evidence-informed decision-making? A qualitative study. Implement Sci.

[21] Dixon-Woods M, Cavers D, Agarwal S, Annandale E, Arthur A, Harvey J (2006). Conducting a critical interpretive synthesis of the literature on access to healthcare by vulnerable groups. BMC Med Res Methodol.

[22] Eakin JM, Mykhalovskiy E (2003). Reframing the evaluation of qualitative health research: reflections on a review of appraisal guidelines in the health sciences. J Eval Clin Pract.

[23] Lindblom CE, Cohen DK. Usable Knowledge: Social Science and Social Problem Solving. New Haven, CT: Yale University Press; 1979.

[24] Majid U, Vanstone M (2018). Appraising qualitative research for evidence syntheses: a compendium of quality appraisal tools. Qual Health Res.

[25] Cohen J (1968). Weighted kappa: Nominal scale agreement provision for scaled disagreement or partial credit. Psychol Bull.

[26] Glaser B, Strauss A. The Discovery of Grounded Theory: Strategies for Qualitative Research. New Jersey: Transaction Publishers; 2009.

[27] Lavis JN, Lomas J, Hamid M, Sewankambo NK (2006). Assessing country-level efforts to link research to action. Bull World Health Organ.

[28] Cochrane. Cochrane Knowledge Translation Strategy. 2017. Cochrane Knowledge Translation Strategy. 2017. https://community.cochrane.org/sites/default/files/uploads/inline-files/Cochrane%20Knowledge%20Translation%20Strategy%20FINAL%20for%20website.pdf. Accessed 31 Jan 2020.

[29] Charmaz K. Constructing Grounded Theory. Thousand Oaks, CA: Sage; 2014.

[30] Reed BA, Habicht JP, Garza C (2002). Translating nutrition research into action in humanitarian emergencies. J Nutr.

[31] Tharyan P, Clarke M, Green S (2005). How the Cochrane collaboration is responding to the Asian tsunami. PLoS Med.

[32] Kutcher S, Chehil S, Roberts T (2005). An integrated program to train local health care providers to meet post-disaster mental health needs. Rev Panam Salud Publica.

[33] Clarke M (2008). Evidence Aid--from the Asian tsunami to the Wenchuan earthquake. J Evid Based Med.

[34] Bornemisza O, Zwi A. Neglected Health Systems Research: Health Policy and Systems Research in Conflict-Affected Fragile States. Geneva: World Health Organization; 2009.

[35] O'Mathuna DP (2010). Conducting research in the aftermath of disasters: ethical considerations. J Evid Based Med.

[36] Roy N, Thakkar P, Shah H (2011). Developing-world disaster research: present evidence and future priorities. Disaster Med.

[37] Aitken P, Leggat PA, Robertson AG, Harley H, Speare R, Leclercq MG (2012). Leadership and use of standards by Australian disaster medical assistance teams: Results of a national survey of team members. Prehosp Disaster Med.

[38] Tol WA, Patel V, Tomlinson M, Baingana F, Galappatti A, Silove D (2012). Relevance or excellence? Setting research priorities for mental health and psychosocial support in humanitarian settings. Harv Rev Psychiatr.

[39] Altay N, Labonte M (2014). Challenges in humanitarian information management and exchange: evidence from Haiti. Disasters..

[40] Gimenez R (2014). Developing a Community of Practice to Learn, Share and Improve in Emergency Management. Eur Conf Knowl Manage.

[41] Knox Clarke P, Darcy J (2014). Insufficient Evidence? The Quality and Use of Evidence in Humanitarian Action (ALNAP Study).

[42] Lee AC, Booth A, Challen K, Gardois P, Goodacre S (2014). Disaster management in low- and middle-income countries: scoping review of the evidence base. Emerg Med J.

[43] Mahapatra P (2014). The need for evidence-based public health response in disasters. J Evid Based Med.

[44] Allen C, Clarke MJ (2015). Evidence Aid: A resource for those preparing for and responding to disasters, humanitarian crises and major healthcare emergencies. Trop Med Int Health.

[45] Kadhiravan T (2015). Why do we need systematic reviews to inform healthcare decisions in the disaster context. J Evid Based Med.

[46] Kayabu B (2015). Evidence Aid approach to gap analysis and priority setting of questions for systematic reviews in disasters. J Evid Based Med.

[47] Mellon D (2015). Evaluating Evidence Aid as a complex, multicomponent knowledge translation intervention. J Evid Based Med.

[48] Mutasa M (2015). Knowledge apartheid in disaster risk management discourse: Is marrying indigenous and scientific knowledge the missing link?. Jamba-J Disaster Risk Stud.

[49] Pottie K (2015). Health equity in humanitarian emergencies: a role for evidence aid. J Evid Based Med.

[50] Reyers B, Nel JL, O'Farrell PJ, Sitas N, Nel DC (2015). Navigating complexity through knowledge coproduction: Mainstreaming ecosystem services into disaster risk reduction. Proc Natl Acad Sci U S A.

[51] Department for International Development. Humanitarian Evidence Systems Mapping in East Africa. 2016. https://www.gov.uk/dfid-research-outputs/humanitarian-evidence-systems-mapping-in-east-africa. Accessed 31 Jan 2020.

[52] Lee ACK (2016). Barriers to evidence-based disaster management in Nepal: A qualitative study. Public Health.

[53] Sitas N, Reyers B, Cundill G, Prozesky HE, Nel JL, Esler KJ (2016). Fostering collaboration for knowledge and action in disaster management in South Africa. Curr Opin Environ Sustain.

[54] Woodward A, Sondorp E, Witter S, Martineau T (2016). Health systems research in fragile and conflict-affected states: a research agenda-setting exercise. Health Res Policy Syst.

[55] Woodward A, Sheahan K, Martineau T, Sondorp E (2017). Health systems research in fragile and conflict affected states: a qualitative study of associated challenges. Health Res Policy Syst.

[56] Fleiss JL, Cohen J, Everitt B (1969). Large sample standard errors of kappa and weighted kappa. Psychol Bull.

[57] Hall PA. The role of interests, institutions, and ideas in the comparative political economy of the industrialized nations. Comp Polit. 1997:174–207.

[58] Pang T, Sadana R, Hanney S, Bhutta ZA, Hyder AA, Simon J (2003). Knowledge for better health: a conceptual framework and foundation for health research systems. Bull World Health Organ.

[59] Lavis JN, Wilson MG, Moat KA, Hammill AC, Boyko JA, Grimshaw JM (2015). Developing and refining the methods for a ‘one-stop shop’for research evidence about health systems. Health Res Policy Syst.

[60] Kapucu Naim, Arslan Tolga, Demiroz Fatih (2010). Collaborative emergency management and national emergency management network. Disaster Prevention and Management: An International Journal.

[61] Kapucu N, Garayev V (2011). Collaborative decision-making in emergency and disaster management. Int J Public Adm.

[62] Gregoire V, Evans M, Le QT, Bourhis J, Budach V, Chen A, et al. Delineation of the primary tumour Clinical Target Volumes (CTV-P) in laryngeal, hypopharyngeal, oropharyngeal and oral cavity squamous cell carcinoma: AIRO, CACA, DAHANCA, EORTC, GEORCC, GORTEC, HKNPCSG, HNCIG, IAG-KHT, LPRHHT, NCIC CTG, NCRI, NRG Oncology, PHNS, SBRT, SOMERA, SRO, SSHNO, TROG consensus guidelines. Radiother Oncol. 2018;126(1):3–24. 10.1016/j.radonc.2017.10.016 29180076

[63] Lavis JN, Robertson D, Woodside JM, McLeod CB, Abelson J (2003). How can research organizations more effectively transfer research knowledge to decision makers?. Milbank Q.

[64] Hermann CF. International Crises. Insights from Behavioral Research. New York, NY: Free Press; 1972.

[65] Boyko JA, Lavis JN, Abelson J, Dobbins M, Carter N (2012). Deliberative dialogues as a mechanism for knowledge translation and exchange in health systems decision-making. Soc Sci Med.

[66] El-Jardali F, Hammoud R, Fouad F, Bou KL. K2P Briefing Note: Promoting Access to Essential Health Care Services for Syrian Refugees in Lebanon. Beirut: Knowledge to Policy (K2P) Center Beirut; 2014. p. 6.

[67] El-Jardali F, Lavis JN, Ataya N, Jamal D (2012). Use of health systems and policy research evidence in the health policymaking in eastern Mediterranean countries: views and practices of researchers. Implement Sci.

[68] Mijumbi RM, Oxman AD, Panisset U, Sewankambo NK (2014). Feasibility of a rapid response mechanism to meet policymakers' urgent needs for research evidence about health systems in a low income country: a case study. Implement Sci.

[69] Ammar W, Kdouh O, Hammoud R, Hamadeh R, Harb H, Ammar Z, et al. Health system resilience: Lebanon and the Syrian refugee crisis. J Glob Health. 2016;6:020704 10.7189/jogh.06.020704 PMC5234495 28154758

[70] Blanchet K, Allen C, Breckon J, Davies P, Duclos D, Jansen J, et al. Research Evidence in the Humanitarian Sector: A Practice Guide. 2018. https://www.alliance4usefulevidence.org/publication/research-evidence-in-the-humanitarian-sector-a-practice-guide/. Accessed 31 Jan 2020.

[71] Ward V, House A, Hamer S (2009). Knowledge brokering: the missing link in the evidence to action chain?. Evid Policy.

[72] Bradt DA. Evidence-based decision-making in humanitarian assistance: HPN Network Paper-Humanitarian Practice Network, Overseas Development Institute; 2009. p. 67.

[73] Garfield R. Common needs assessments and humanitarian action: Humanitarian Practice Network: Overseas Development Institute; 2010.

[74] Jillson IA, Clarke M, Allen C, Waller S, Koehlmoos T, Mumford W (2019). Improving the science and evidence base of disaster response: a policy research study. BMC Health Serv Res.

[75] Lomas Jonathan (2007). The in-between world of knowledge brokering. BMJ.

[76] Lavis JN, Boyko JA, Oxman AD, Lewin S, Fretheim A (2009). SUPPORT Tools for evidence-informed health Policymaking (STP) 14: Organising and using policy dialogues to support evidence-informed policymaking. Health Res Policy Syst.

[77] Lomas J. Improving Research Dissemination and Uptake in the Health Sector: Beyond the Sound of One Hand Clapping. Hamilton: Centre for Health Economics and Policy Analysis; 1997.

[78] Chalmers I (2005). If evidence-informed policy works in practice, does it matter if it doesn't work in theory?. Evid Policy.

[79] Straus SE, McAlister FA. Evidence-based medicine: a commentary on common criticisms. CMAJ. 2000;163(7):837–41. PMC80509 11033714

[80] Clarence Emma (2002). Technocracy Reinvented: The New Evidence Based Policy Movement. Public Policy and Administration.

[81] Parsons W (2002). From muddling through to muddling up-evidence based policy making and the modernisation of British Government. Public Policy Admin.

[82] Blanchet K, Roberts B, Sistenich V, Ramesh A, Frison S, Warren E, et al. An Evidence Review of Research on Health Interventions in Humanitarian Crises. Final Report November 2013. London: London School of Hygiene and Tropical Medicine; 2013.

[83] Lavis JN, Boyko JA, Gauvin F-P (2014). Evaluating deliberative dialogues focussed on healthy public policy. BMC Public Health.

[84] Ellen ME, Léon G, Bouchard G, Ouimet M, Grimshaw JM, Lavis JN (2014). Barriers, facilitators and views about next steps to implementing supports for evidence-informed decision-making in health systems: a qualitative study. Implement Sci.

[85] Khangura S, Konnyu K, Cushman R, Grimshaw J, Moher D (2012). Evidence summaries: the evolution of a rapid review approach. Syst Rev.

[86] Chambers D, Wilson PM, Thompson CA, Hanbury A, Farley K, Light K (2011). Maximizing the impact of systematic reviews in health care decision making: a systematic scoping review of knowledge-translation resources. Milbank Q.

