# Supplementary material for: Supporting the use of research evidence in decision-making in crisis zones in low- and middle-income countries: a critical interpretive synthesis
Source: Health Res Policy Syst. 2020 Feb 18;18:21. doi: 10.1186/s12961-020-0530-2 (PMC7027202; doi:10.1186/s12961-020-0530-2)
Supplement: Supplementary file 1 — Additional file 1: Table S1. Initial database search strategy. [file 12961_2020_530_MOESM1_ESM.docx]

**Additional File 1**

**Table S1**. Initial database search strategy

| **Synonyms for knowledge translation** | **AND** | **Synonyms for crisis** | **AND** | **Synonyms for LMIC** |
| --- | --- | --- | --- | --- |
| Knowledge*  (meant to capture knowledge translation, knowledge synthesis, knowledge, dissemination, knowledge exchange, knowledge transfer) |  | Cris*  (meant to capture crisis, crises) |  | Norwegian satellite EPOC filter  (meant to capture all related terms to LMIC and list of LMIC countries) |
| Evidence*  (meant to capture evidence, evidence-based, evidence-informed, evidence-informed policy, evidence based decision making) |  | Emergency |  | **1.** Developing Country**.sh.**  **2.** (Africa or Asia or Caribbean or West Indies or South America or Latin America or Central America)**.hw,ti,ab,cp.**  **3.** (Afghanistan or Albania or Algeria or Angola or Antigua or Barbuda or Argentina or Armenia or Armenian or Aruba or Azerbaijan or Bahrain or Bangladesh or Barbados or Benin or Byelarus or Byelorussian or Belarus or Belorussian or Belorussia or Belize or Bhutan or Bolivia or Bosnia or Herzegovina or Hercegovina or Botswana or Brasil or Brazil or Bulgaria or Burkina Faso or Burkina Fasso or Upper Volta or Burundi or Urundi or Cambodia or Khmer Republic or Kampuchea or Cameroon or Cameroons or Cameron or Camerons or Cape Verde or Central African Republic or Chad or Chile or China or Colombia or Comoros or Comoro Islands or Comores or Mayotte or Congo or Zaire or Costa Rica or Cote d'Ivoire or Ivory Coast or Croatia or Cuba or Cyprus or Czechoslovakia or Czech Republic or Slovakia or Slovak Republic or Djibouti or French Somaliland or Dominica or Dominican Republic or East Timor or East Timur or Timor Leste or Ecuador or Egypt or United Arab Republic or El Salvador or Eritrea or Estonia or Ethiopia or Fiji or Gabon or Gabonese Republic or Gambia or Gaza or Georgia Republic or Georgian Republic or Ghana or Gold Coast or Greece or Grenada or Guatemala or Guinea or Guam or Guiana or Guyana or Haiti or Honduras or Hungary or India or Maldives or Indonesia or Iran or Iraq or Isle of Man or Jamaica or Jordan or Kazakhstan or Kazakh or Kenya or Kiribati or Korea or Kosovo or Kyrgyzstan or Kirghizia or Kyrgyz Republic or Kirghiz or Kirgizstan or Lao PDR or Laos or Latvia or Lebanon or Lesotho or Basutoland or Liberia or Libya or Lithuania or Macedonia or Madagascar or Malagasy Republic or Malaysia or Malaya or Malay or Sabah or Sarawak or Malawi or Nyasaland or Mali or Malta or Marshall Islands or Mauritania or Mauritius or Agalega Islands or Mexico or Micronesia or Middle East or Moldova or Moldovia or Moldovian or Mongolia or Montenegro or Morocco or Ifni or Mozambique or Myanmar or Myanma or Burma or Namibia or Nepal or Netherlands Antilles or New Caledonia or Nicaragua or Niger or Nigeria or Northern Mariana Islands or Oman or Muscat or Pakistan or Palau or Palestine or Panama or Paraguay or Peru or Philippines or Philipines or Phillipines or Phillippines or Poland or Portugal or Puerto Rico or Romania or Rumania or Roumania or Russia or Russian or Rwanda or Ruanda or Saint Kitts or St Kitts or Nevis or Saint Lucia or St Lucia or Saint Vincent or St Vincent or Grenadines or Samoa or Samoan Islands or Navigator Island or Navigator Islands or Sao Tome or Saudi Arabia or Senegal or Serbia or Montenegro or Seychelles or Sierra Leone or Slovenia or Sri Lanka or Ceylon or Solomon Islands or Somalia or South Africa or Sudan or Suriname or Surinam or Swaziland or Syria or Tajikistan or Tadzhikistan or Tadjikistan or Tadzhik or Tanzania or Thailand or Togo or Togolese Republic or Tonga or Trinidad or Tobago or Tunisia or Turkey or Turkmenistan or Turkmen or Uganda or Ukraine or Uruguay or USSR or Soviet Union or Union of Soviet Socialist Republics or Uzbekistan or Uzbek or Vanuatu or New Hebrides or Venezuela or Vietnam or Viet Nam or West Bank or Yemen or Yugoslavia or Zambia or Zimbabwe or Rhodesia).**hw,ti,ab,cp.**  **4.** ((developing or less* developed or under developed or underdeveloped or middle income or low* income or underserved or under served or deprived or poor*) adj (countr* or nation? or population? or world))**.ti,ab.**  **5.** ((developing or less* developed or under developed or underdeveloped or middle income or low* income) adj (economy or economies))**.ti,ab.**  **6.** (low* adj (gdp or gnp or gross domestic or gross national))**.ti,ab.**  **7.** (low adj3 middle adj3 countr*)**.ti,ab.**  **8.** (lmic or lmics or third world or lami countr*)**.ti,ab.**  **9.** transitional countr***.ti,ab.**  **10.** or/1-9 |
| Research*  (meant to capture research, research-based, research informed) |  | Disaster |  |  |
| Scien*  (meant to capture science, scientific, scientist) |  | Catastrophe |  |  |
| Guideline |  | Conflict |  |  |
| Guideline Adherence |  | War |  |  |
| Program Evaluation |  | “Natural hazard” |  |  |
| Evidence-Based Practice |  | “Geophysical hazard” |  |  |
|  |  | “Hydrological hazard” |  |  |
|  |  | “Climatological hazard” |  |  |
|  |  | “Meteorological hazard” |  |  |
|  |  | “Biological hazard” |  |  |
|  |  | “Man-made hazard” |  |  |
|  |  | “Technological hazard” |  |  |
|  |  | Famine |  |  |
|  |  | “Displaced population” |  |  |
|  |  | “Industrial accident” |  |  |
|  |  | “Transport accident” |  |  |
|  |  | Earthquake |  |  |
|  |  | Landslide |  |  |
|  |  | Tsunamis |  |  |
|  |  | Volcan* |  |  |
|  |  | Avalanche |  |  |
|  |  | Flood |  |  |
|  |  | “Extreme temperature” |  |  |
|  |  | Drought |  |  |
|  |  | Wildfire |  |  |
|  |  | “Cyclone surge” |  |  |
|  |  | “Storm surge” |  |  |
|  |  | “Wave surge” |  |  |
|  |  | “Disease epidemic” |  |  |
|  |  | “Insect plague” |  |  |
|  |  | “Animal plague” |  |  |
